# Remote work arrangement: a blessing in disguise for socially anxious individuals

**DOI:** 10.3389/fpsyg.2023.1152499

**Published:** 2024-05-16

**Authors:** Khalid Khan, Umm e-Habiba, Shahab Aziz, Zara Sabeen, Asma Zeeshan, Zareen Naz, Muhammad Waseem

**Affiliations:** ^1^Department of Business Administration, Federal Urdu University of Arts, Sciences and Technology Islamabad, Islamabad, Pakistan; ^2^Department of Management Sciences, Bahria University Islamabad, Islamabad, Pakistan; ^3^Interdisciplinary Research Center (IRC) for Finance and Digital Economy, KFUPM Business School, King Fahd University of Petroleum and Minerals (KFUPM), Dhahran, Saudi Arabia; ^4^Department of Islamic Art and Architecture, International Islamic University Islamabad, Islamabad, Pakistan; ^5^Department of Management Studies, Bahria Business School, Bahria University, Islamabad, Pakistan

**Keywords:** social anxiety disorder (SAD), psychological distance (PD), burnout, attachment orientation, PLS-SEM

## Abstract

This research examines the link between social anxiety disorder (SAD), psychological distance (PD), and burnout using survey data from 463 software development workers who are currently working remotely. According to the results of the study, SAD was associated with higher PD, but, in contrast to what had been shown in earlier studies, this higher PD had no effect on the participants' reported levels of burnout. Both psychological safety and workplace attachment orientation (WAO) were tested for their moderating effects in this study. According to the study's findings, the link between SAD and PD was moderated by WAO but not by psychological safety. The findings of this study underscore the importance of conducting additional research on the challenges faced by people with social anxiety in the workplace and the steps that can be taken by employers to better accommodate them.

## 1. Introduction

The foundation of any modern organization is the interpersonal networks of its members. When it comes to building and maintaining professional networks, individuals are influenced by their social exchange relationships. An individual's likelihood of forming a relationship with co-workers whom they perceive to be able to provide them with an advantage increases if they believe that these co-workers have access to knowledge and/or resources that will be useful to them. The quality of these relationships can have an effect on the chances for promotion and advancement within an organization. Social aptitude, defined as “one's innate talent or competence in dealing with people”, varies greatly from person to person (Saluck, 2006). The ability to connect with others, understand and use non-verbal clues, and read and respond appropriately to emotional cues are all examples of the kinds of talents and attributes that fall under the umbrella term of “social aptitude” (Albrecht et al., 1982).

Individuals with certain inclinations can become socially incompetent. Social anxiety disorder is an important precursor of social ineptness. A socially anxious individual (SAI) is concerned about how others will see him or her (Levinson et al., 2014). This dread generates behaviors that are met with unfavorable responses from interaction partners, reinforcing SAI's negative perceptions. Previous research has found that SAIs have low quality and quantity of social interactions (Whisman et al., 2000; Chou et al., 2011). Because of their social ineptness, SAIs are unable to benefit from social support from various organizational foci within the organizational setting.

Positive social connections (Heaphy and Dutton, 2008) have an important role in physically and psychologically bringing interaction partners closer together. The current study uses psychological distance as a measure of an interaction partner's perceived proximity to a focal person (Trope and Liberman, 2010). SAIs are fearful of negative reactions from others, which is why they keep a greater physical distance between themselves and their interaction partners (Wieser et al., 2010). Their actions also distance them from their interaction partners. SAIs also fear that people will judge them harshly; therefore, they view their interaction partners to be psychologically distant from them (Clark and Adrian, 1995).

Remote working provides another layer to the perceived distance between interaction partners (Hayes et al., 2020). SAIs are sensitive to social cues from their interaction partners (Schmidt et al., 2009), and communicating via video conferencing or messaging diminishes their ability to gather these data. Remote working also heightens the SAI's sense of loneliness, which already puts them at a disadvantage when it comes to making social connections (Boucher et al., 2021). Finally, one additional problem that SAIs experience while working remotely is establishing limits for when and how they are available to communicate with others, as there is perceived pressure to be always available.

The challenges associated with remote working highlighted above can be mitigated by providing a psychologically safe working environment. Such an environment encourages experimentation and allows the interaction partners to express themselves without the fear of reprisal or any other negative consequences (Newman et al., 2017). Psychological safety provides the platform for interpersonal relationships that are based on interpersonal trust, mutual respect, and confidence in each other's competence (Edmondson, 1999). Such a positive environment can become instrumental for an SAI to overcome his/her social phobias. A psychologically safe environment would also facilitate in the reduction of the perceived psychological distance between the interaction partners (Huang, 2015). A relationship of trust and respect brings the interaction partners physically closer and allows the formation of mental models of others where the others are seen as reliable sources of support.

Individuals also vary in their ability to form meaningful social relationships based on their attachment orientation (McCarty, 2005). Attachment orientation refers to an individual's characteristic style of attachment or the way in which they relate to others in emotional and physical proximity (Mikulincer et al., 2021). The attachment literature distinguishes two types of attachment styles: anxious attachment and avoidant attachment. Anxious attachment is characterized by a preoccupation with the availability and responsiveness of others, a need for constant reassurance and validation from others, and a fear of abandonment. Avoidant attachment is characterized by a desire for independence and self-reliance, a discomfort with emotional intimacy and dependence on others, and a tendency to suppress or avoid the expression of emotions (Martin et al., 2022).

Research on remote working has reported on the many positive aspects of this form of work. Nevertheless, there are findings that allude to remote workers facing loneliness and burnout (Moss, 2018). SAIs working remotely are at a further disadvantage as this reduces their ability to have meaningful social interactions with their interaction partners. The aim of the present study was to model the direct and indirect effects of SAD on work-related burnout. The indirect effect was tested using psychological distance as a mediator. The theoretical model also tested for the moderation effects of psychological safety and attention orientation on the effect of SAD on psychological distance.

## 2. Literature review

### 2.1. Social anxiety disorder

Social anxiety disorder (SAD) was recognized as a mental disorder in 1980 by the American Psychiatric Association (Wong et al., 2014). Since then, theories have emerged trying to explain the cognitive and behavioral aspects of SAD. Wong et al. (2014) provide an excellent review of the various cognitive-behavioral models of SAD; for the sake of brevity, this discussion will not be reproduced here. The present review will focus on the interpersonal model of SAD which postulates that socially anxious individuals form negative expectations about socialization based on their past social experiences, biological factors, and relational schemas consisting of models of self and others (Wong et al., 2014).

Alden et al. (2014) summarize that individuals with SAD have significant social deficiencies. Furthermore, that these deficiencies negatively affect physical and emotional wellbeing. Studies have shown that SAIs have smaller social networks in real life as well as using online social media platforms (Tian, 2013). Research also suggests that not only do SAIs have small social networks, but they also tend to have fewer interactions with those in their social networks (Oren-Yagoda and Aderka, 2021; Oren-Yagoda et al., 2021). Studies have also shown that SAIs tend to be lonelier, have fewer friends, and are less likely to participate in social groupings (Falk Dahl and Dahl, 2010).

A logical consequence of social ineptness, manifested by SAD, is that interaction partners find SAIs lacking in different aspects of life. Anderson et al. (2015) found similarities between how individuals with mental health issues, those suffering from major depressive disorder, and SAIs were stigmatized. Their study showed that interaction partners preferred more social distance from SAIs because the interaction partners viewed them as dangerous and as being inflicted with an embarrassing problem. Specific to this study, the views regarding SAIs differed among the observers who viewed SAD as being either avoidable, common among women, or the possibility of it being a source of work-related problems.

Studies on SAD have shown that the dysfunctional social behaviors attributed to SAD by the interaction partners can be safety behaviors adopted by the SAIs (Alden et al., 2014). Alden et al. (2014, p. 167) define safety behaviors as “as overt or covert acts intended to manage or avert a perceived threat and increase the person's sense of safety”. Safety behaviors that are commonly adopted include avoidance and impression management. The actions associated with the avoidance behavior include avoiding direct eye contact, maintaining a low profile, and limiting speech (Alden et al., 2014). Impression management is intended to disguise one true self by controlling one's natural behaviors, faking facial expressions, and practicing saying what others want to hear but not what one wants to say. Alden et al. (2014) also note that when choosing from the various safety behaviors the SAIs make a strategic choice. And that these choices sometimes result in negative reactions because the SAIs focus becomes occupied with these behaviors rather than taking a genuine interest in the interaction. Previous studies (Plasencia et al., 2011; Taylor and Alden, 2011) have shown that interaction partners tend to react negatively to both avoidance and impression management behaviors.

Several studies have been conducted to understand the inability of SAIs to establish meaningful relationships (Alden et al., 2014). Reis and Shaver's (1988) model has been the foundation for a significant number of these studies. This model stipulates that intimate relationships are based on two important aspects: self-disclosure and responsiveness. Self-disclosure occurs when interaction partners disclose something personal about themselves, which was previously not known by the other party to the interaction (Alden et al., 2014). Responsiveness is a measure of how interaction partners attend to support each other's needs and goals (Reis and Gable, 2015). Alden et al. (2014) concluded, after a review of the relevant literature, that SAIs display shortcomings in both self-disclosure and responsiveness, thus contributing to their overall social deficiencies.

In conclusion, the above summary provides the current available evidence on how the social deficiencies attributed to SAIs causes them to struggle to maintain meaningful social connections. The review also informs about how others respond negatively to the behaviors commonly attributed to SAIs. Based on the above literature, it can be concluded that SAIs will also face a difficult time working in an organizational setting where social networks are considered to be an important resource (Bassett and Moore, 2013; Mo et al., 2014). This challenge has further been exacerbated when one must work remotely. Remote work adds to the physical and psychological distance between interaction partners. The following section of the literature review describes the construct of psychological distance in greater detail.

### 2.2. Psychological distance

The origins of psychological distance (PD) can be traced back to the temporal level theory (Chen and Li, 2018). The temporal construal theory holds that people's responses to past, present, and future events vary depending on how close or far they are from the event. Support for this temporal effect of time can be found in the works of Kahneman (2011) who lists several behavioral phenomena including the recency bias where individuals remember recent events better than older ones. Evidence for the temporal effect can also be drawn from the phenomenon described as “hedonic treadmill.” The term was first coined by Philip Brickman and Donald T. Campbell in 1971, to describe the tendency of individuals to compare present life events to temporally proximal states of their lives, rather than how they felt at a temporally distant moment. Similarly, Thaler and Sunstein (2008) note that “[R]ecent events have a greater impact on our behavior, and on our fears, than earlier ones”.

Liberman et al. (2007) contributed toward the operationalization of the construct of psychological distance by including the aspects of physical distance, social distance, and the probability of occurrence. They argue that individuals form different levels of abstractions about events depending on the psychological distance from the event. Individuals form low-level abstractions of events that are psychologically near and high-level abstractions of events that are far. Consider the distinction between an individual who has a significant other who has been inflicted with SAD and an individual who does interact with individuals suffering from SAD but is not aware of their condition. The former will recognize the symptoms of SAD in other individuals and thus would have a low-level abstraction of SAD. This individual would be more conscious of the behavioral cues associated with SAD. The person who is not aware of SAD might simply categorize the symptoms associated with SAD as social awkwardness (Lipsitz, 2006). This individual would have formed high-level abstractions about those inflicted with SAD.

Building on the construct of psychological distance, researchers within the field of organizational studies have started to operationalize the perceived psychological distance between various organizational foci. For example, Chen and Li (2018) postulate that workplace behaviors are shaped by the construal representation of the organization held by the employees of the organization. This construal representation of the organization, according to Chen and Li (2018), depends on the organizational context and the idiosyncratic attributes of the focal person. They further note that the social exchanges prevailing between the various organizational foci and a focal person are also shaped by the perceived psychological distance between them.

Chen and Li (2018) offer a multifaceted operationalization of the employee–organization psychological distance. The first of these facets being temporal distance which is measured as the duration that the focal person has remained attached to the organization. Second, the spatial distance which is a measure of the physical distance between the focal person and the organization. The relevance of this facet has increased since the onset of the COVID pandemic which has made remote working—working away from the physical premises of the organization—a reality for a greater number of people than before the pandemic. The third facet operationalized by Chen and Li (2018) is social distance which they describe as the quality of social relationships of the focal person with other organizational foci. The remaining two facets include expectations—about future changes that may unfold within the organization—and emotional belonging (a measure of extent to which values are shared across the organization). The latter two facets identified by Chen and Li (2018) are operationalized as measures of a focal person's affinity for the organizations and are not included in the measures of distance. Thus, for the purposes of the current study psychological distance is operationalized as consisting of the facets of temporal, spatial, and social distance.

### 2.3. Occupational burnout

After an analysis of more than 88 unique definitions of occupational burnout present in the literature and using a panel of 50 experts, Bakker et al. (2002) provide the following definition of occupational burnout “In a worker, occupational burnout or occupational physical AND emotional exhaustion state is an exhaustion due to prolonged exposure to work-related problems” (p. 104). Although the literature provides assorted definitions of the construct, there is no disagreement regarding the negative mental and physical outcomes experienced because of burnout. It is due to this strong evidence that WHO declared occupational burnout as a major health issue in the International Classification of Diseases 11th revision (ICD-11, Woo et al., 2020).

The extant literature identifies occupational burnout as a multifaceted construct with the following three dimensions: emotional exhaustion (Jarzynkowski et al., 2021; Xie et al., 2021; Shbeer and Ageel, 2022), depersonalization/cynicism (Clifton et al., 2021; Jarzynkowski et al., 2021; Shbeer and Ageel, 2022), and inefficacy (Khosravi, 2021; Khosravi et al., 2022; Zaluski and Makara-Studzińska, 2022). A focal person normally feels emotionally exhausted when faced with an emotionally taxing environment where one has to portray emotions different than those that are felt (Lozano, 2021). Faced with an increasing sense of emotional exhaustion, individuals generally tend to become indifferent toward their work and co-workers to reduce the chance of further emotional exhaustion (Niu et al., 2022). This indifference leads to depersonalizations or cynicism, which has counterproductive effects on a focal person's performance (Maslach et al., 2001). This drop in performance intensifies a focal person's feeling of inefficacy, which singles a loss of confidence in one's ability to adequately perform the task assigned to him/her (Angerer, 2003).

Burnout is typically experienced when an individual is overworked, i.e., when a focal person is assigned quantitatively large amount of work whereby the focal person becomes overstretched (Lee and Ashforth, 1996). Role-related constructs such as role conflict and role ambiguity have also been linked to work-related burnout (Schwab and Iwanicki, 1982). Role conflict occurs when conflicting demands are placed on the role occupant (Schaufeli and Buunk, 2003). Whereas, role ambiguity becomes a problem when the occupant of a role is not provided with the necessary information to perform the role assigned to him/her. Schaufeli and Buunk (2003) identified three causes of burnout: lack of social support, lack of self-regulatory authority, and client-related demands. Social support from supervisors and co-workers can help minimize burnout, but the findings in this regard are equivocal. Self-regulation, allowing for decision-making and providing feedback, can also help counter burnout. Difficult clients can also contribute to burnout but to a lesser extent.

### 2.4. Psychological safety

Psychological safety (PS) is a measure of the willingness of individuals to take interpersonal risks in a group setting (Edmondson, 1999). Edmondson (1999), while distinguishing between the constructs of general trust and PS, elaborates that the conceptualization of the PS has more to do with group norms and beliefs rather than interpersonal trust. As such, PS is an evaluation of the group climate to encourage experimentation and voice without the fear of reprisal or other consequences. Edmondson (1999) operationalizes PS as a blend between interpersonal trust, mutual respect, and confidence in each other's competence.

Shain et al. (2012) conducted a study to determine the work-related antecedents of PS. The results from their study indicate that perceived psychological safety is affected by the perceptions of whether the task assigned to individuals is within their capacity. Individuals who were offered a choice of tasks to perform were more likely to report better psychological safety. Psychological safety is also improved when rewards are provided in a timely manner and due process is followed. Shain et al. (2012) found that a person's sense of psychological safety decreases when the members of their group withhold information that was crucial for completing tasks.

Erkutlu and Chafra (2016) discovered that the absence of PS can have a detrimental impact on an individual's mental health. In groups that offer a low level of PS, the members are prone to suppressing their voices and concealing their real thoughts. Additionally, these members are less likely to request additional resources that they need to complete tasks. Lastly, members of groups with low PS are more likely to overlook problems instead of bringing them to attention.

Newman et al. (2017) similarly found that psychological safety (PS) can have a negative impact on an individual's wellbeing and mental health. This conclusion was based on a comprehensive review of the literature on PS. Early research on PS was primarily conducted in the field of sports and exercise sciences and focused on the mental health of professionals in these areas (Gouttebarge et al., 2019). More recently, the concept of PS has been adopted by studies that examine the performance of work-related teams, suggesting that creating a work environment that promotes PS can have a positive effect on the mental health and wellbeing of team members, leading to improved performance (Newman et al., 2017).

The existing literature on psychological safety (PS) has noted several positive outcomes associated with PS, including increased creativity (Carmeli et al., 2010), proactive involvement in group tasks (Yi et al., 2017), increased workplace engagement (Frazier et al., 2017), reduced anxiety (Hur et al., 2018; Ahmad et al., 2019), a willingness to provide and receive positive feedback (Newman et al., 2017), improved learning ability (Edmondson, 1999), and the desire to express one's true self (Kark and Carmeli, 2009). Kim and Kim (2020) also found in their study on third-party justice (a measure of how fairly an organization treats external parties) that positive perceptions of third-party justice have a positive impact on PS.

### 2.5. Attachment orientation

The construct of attachment orientation is used to explain how a person's prior experiences with important figures in their life impact their future social relationships (Brazeau and Chopik, 2020). This construct is rooted in attachment theory, which looks at the quality of social interactions. According to this theory, people can fall anywhere on a spectrum from having an insecure attachment orientation to having a secure attachment (Read et al., 2018). This differentiation between individuals is based on their scores on two aspects of attachment orientation: attachment anxiety and attachment avoidance. Attachment anxiety assesses the amount of fear a person feels about being rejected or abandoned by someone they are attached to Vowels et al. (2022), while attachment avoidance measures the level of discomfort a person feels about becoming emotionally attached or dependent on others (Bayraktaroglu et al., 2022).

According to Read et al. (2018), individuals with an insecure attachment orientation may employ one of two types of attachment strategies, based on whether they are struggling with high levels of attachment anxiety or attachment avoidance. Those who experience high attachment anxiety are likely to adopt hyperactivating strategies, such as excessive clinginess, forceful behavior, and over-reliance on their relationship partners (Civilotti et al., 2021; Sood et al., 2022). However, those with attachment avoidance tend to use deactivating attachment strategies, such as avoiding emotional involvement, becoming more self-sufficient, and suppressing their feelings of attachment (Martin et al., 2022), to reduce the perceived harm that comes from forming attachments.

In organizational psychology research, there is now a shift beyond examining how attachment orientation affects interpersonal relationships and instead a recognition of workplace attachment as its own separate construct (Scrima et al., 2017). This idea of workplace attachment is rooted in the concept of place attachment, which describes how people form attachments to physical spaces they have a connection to Inalhan et al. (2021). There are three major models of workplace attachment found in the literature, including Rioux's (2006) unidimensional model, the person–process–place model that views workplace attachment as a multidimensional construct, and the workplace attachment-and-disruption model which suggests that workplace attachment only becomes apparent when an individual experiences separation from the workplace.

Previous studies have shown that workplace attachment can result in a reduction in turnover (Chen et al., 2021), is a better predictor of the willingness to separate from a location (apply for a transfer) than workplace attachment and professional life satisfaction (Rioux and Penner, 2001), can motivate organizational citizenship behavior (Rioux and Pavalache-Ilie, 2013), and can improve work engagement (Mura et al., 2023). Studies have shown that individuals with high office attachment (a form of workplace attachment) exhibit a form of territorial behavior and that these individuals can be negatively affected by any changes to their office space (Frankó et al., 2022). Additionally, Nappi-Choulet et al. (2019) reported that workplace attachment mediated the relationship between workspace satisfaction and workplace effectiveness of remote work.

### 2.6. Social anxiety disorder and psychological distance

A defining attribute of socially anxious individuals (SAI) is their aversion to social interactions (Kashdan et al., 2013). Due to this peculiarity, individuals with SAD tend to have a lower frequency of social interactions as compared to individuals without SAD (Oren-Yagoda and Aderka, 2021; Oren-Yagoda et al., 2021). Research conducted on rats with special genetic markers known as Fawn-Hooded—results from laboratory studies have shown that these mice are genetic equivalents of social phobia—indicates that SAD-inflicted individuals tend to have shorter social interaction times as compared to socially abled individuals (Kantor et al., 2000; Boudjafad et al., 2022). Lastly, socially anxious individuals are more likely to maintain a greater interpersonal distance due to their aversion to physical closeness (Givon-Benjio et al., 2020).

Others perceive SAI's inability to form meaningful social connections as a deficit in their social skills (Kelly-Turner and Radomsky, 2022). Individuals with SAD are stigmatized as a result of their perceived differences (Link and Phelan, 2001), which further erodes their ability to bridge the psychological distance between themselves and others (Albrecht et al., 1982). These stigmas associated with SAD contribute to the prevalence of stereotypes associated with people who lack social competence (Anderson et al., 2015). This study on the stigmas associated with SAD concludes that not only does SAI push others away (Thompson and Bunderson, 2003), but others also keep a distance from such people due to the stigmas associated with their illness.

Chen and Li (2018) identify three dimensions of psychological distance between employees and organizations. The first of these is the temporal distance, which describes the duration of the relationship between the focal person and the organizational foci. According to research on social relationships, strong social bonds are formed over time and are dependent on the tenor of the relationship (Silk et al., 2013). Hinde and his colleagues' observations of social relationships among primates support this line of evidence (Hinde and Atkinson, 1970; Hinde and White, 1974). These studies show that the behavior of biologically related entities shapes the quality of social relationships over time. The evidence presented above about socially anxious people suggests that these people will struggle to form long-term relationships because of their own and other's perceptions of their social incompetence.

The second dimension identified by Chen and Li (2018) corresponds to the psychical distance between the interaction partners. The length of distance between interaction partners determines the comfort level between them. Studies have attempted to quantify and categorize various lengths of physical distances maintained between individuals and the type of relationship that can correspond with each distance. Huang (2015) created a classification of relationships based on levels of interpersonal distance, including 0–45 cm (intimate distance), 45–120 cm (personal distance), 120–360 cm (common distance), and >360 cm (public distance). Recently, research has utilized virtual reality (VR) to study the effects of a social anxiety disorder (SAD). These studies have been found to provide valid and reliable results (Wieser et al., 2010). In one study (Wieser et al., 2010), the researchers discovered that levels of avoidance varied in individuals with SAD and were influenced by factors such as the participant's gender, the gender of avatars in the VR scenario, and the intensity of gaze directed at the participant by the avatars.

The last aspect of the psychological distance between employees and their organization is related to social distance. Chen and Li (2018) attributed this to the individual's level of empathy toward others. Empathy can be divided into two types: affective empathy and cognitive empathy (Pittelkow et al., 2021). Affective empathy measures a person's ability to feel the emotions of others (Sowden et al., 2022), and research has shown that this happens when there is an overlap in brain activity areas between people who are reporting on the emotions of others and their own similar emotions (Coll et al., 2017). Cognitive empathy measures a person's ability to understand others' perspectives (Pittelkow et al., 2021). Studies about the relationship between social distance and empathy have produced mixed results. Some studies have found a negative relationship between social distance and empathy, as individuals with high social anxiety may focus more on themselves and ignore the feelings of others (O'Toole et al., 2013), or may over-report negative experiences and therefore have a reduced ability to recognize the emotions of others (Cohen et al., 2017). However, other research suggests that social distance can lead to an increase in empathy (Pittelkow et al., 2021). Considering the above lines of evidence, it is hypothesized that:

*H1*: SAD will lead to an increased level of psychological distance.

### 2.7. Moderating effect of psychological safety

Studies have shown that individuals with social anxiety disorder (SAI) benefit from being in a psychologically safe environment (Brigman et al., 2012). This type of environment encourages people to share their opinions and ideas without fear of judgment or discrimination (Schwappach and Richard, 2018). Teams that foster psychological safety also support their members to learn and grow, and the positive behaviors of their interaction partners, such as acts of kindness, can help to reduce anxiety levels and create a positive perception of social relationships (Clark, 2020). Research indicates that this type of environment is essential for individuals with SAI to overcome their social deficits (see for example, Lyubomirsky and Della Porta, 2010).

According to recent studies by Eisenberg and DiTomaso (2021) and He et al. (2022), psychological safety plays a mediating role in the relationship between social exchange (LMX, supervisor–subordinate guanxi) and work-related outcomes (knowledge-hiding behavior) and between psychological distance and team structures, respectively. These findings suggest that a psychologically safe environment can help reduce the psychological distance between people, resulting in the formation of secure attachments and increased trust. Itzchakov et al. (2016) also found that attentive and non-judgmental listening can improve perceptions of psychological safety. Based on these studies, it can be hypothesized that.

H2: The presence of a psychologically safe work environment will mitigate the indirect effect of social anxiety disorder on burnout, as mediated by psychological distance.

### 2.8. Moderating effect of attachment orientation

According to Team (2022), organizations can alleviate the impact of SAD on their employees by creating a safe and accepting work environment. Indicators of such a work environment include the absence of pressure to participate in social events (Weidman and Levinson, 2015), availability of flexible work arrangements (Mellifont et al., 2016), and opportunities to discuss mental health (Grant, 2017). These types of work settings can help employees with SAD form a secure attachment to their workplace.

According to Read et al. (2018), there is evidence that attachment orientation may play a role in the development of social anxiety. People with an anxious attachment style may be more susceptible to developing social anxiety due to their fear of abandonment and need for constant validation from others (Eng et al., 2001), leading to heightened sensitivity to social cues and fear of rejection or judgment. Conversely, those with an avoidant attachment style may be less prone to developing social anxiety as they tend to suppress their emotions and steer clear of close relationships (Sethia and Markandey, 2022).

The psychological distance people choose to keep between themselves and their interaction partners is thought to be influenced by their attachment orientation (Wang et al., 2012). People who have a stable attachment orientation can create strong, solid ties with others and are at ease with intimacy and reliance, making them well-suited to maintain a healthy level of psychological distance. Individuals with an anxious attachment orientation, however, could find it more difficult to maintain a psychological distance because they might dread abandonment and crave ongoing validation from others. Due to their tendency to repress their feelings and shy away from intimacy in their relationships, people with an avoidant attachment orientation may also struggle to maintain a psychological distance.

However, SAIs with various attachment inclinations may use distinct unique coping mechanisms to preserve psychological distance. For instance, those who are nervous about connection and have social anxiety may strive to keep psychological distance from others by always needing their assurance and steering clear of stressful or conflict-provoking situations (Winterheld, 2016). However, people who have a social anxiety disorder and an avoidant attachment orientation may try to keep psychological distance by repressing their feelings and avoiding intimacy in their interactions (Wang et al., 2012).

There is evidence that attachment orientation may moderate the association between psychological distance and social anxiety. Because they can create solid, safe ties with others and are at ease with intimacy and reliance, people who have a secure attachment orientation may be more likely to maintain a healthy level of psychological distance (McCarty, 2005). Individuals with an anxious attachment orientation, however, can find it more difficult to maintain psychological distance because they might dread abandonment and require ongoing reassurance from others. Due to this dread, they may become highly perceptive of social signs and experience overwhelming anxiety when interacting with others.

Individuals with an anxious attachment orientation and social anxiety may experience more severe symptoms of social anxiety and may have a harder time maintaining psychological distance because of their fear of abandonment and need for reassurance. However, individuals with an avoidant attachment orientation and social anxiety may experience less severe symptoms of social anxiety and may be more able to maintain a psychological distance because of their tendency to suppress their emotions and avoid intimacy in their relationships. Considering the above evidence, it is hypothesized that individuals with an anxious attachment orientation and social anxiety may have more severe social anxiety symptoms and a more difficult time maintaining psychological distance due to their fear of abandonment and need for reassurance. Individuals with an avoidant attachment orientation and social anxiety, however, may experience less severe symptoms of social anxiety and may be better able to maintain psychological distance in their relationships due to their tendency to suppress their emotions and avoid intimacy in their relationships. Based on the facts presented above, it is hypothesized that:

H3: Attachment orientation will moderate the association between SAD and burnout as mediated by psychological distance.

### 2.9. Psychological distance and burnout

Employee–organization psychological distance, according to Chen and Li (2018), is a reflection of the employee–organization relationship. The result is that perceived proximity to the organization is associated with positive behavioral outcomes such as reduced inclination to quit (Yalabik et al., 2017). Occupational burnout, according to Chen and Li (2018), is also a reflection of the greater psychological distance between employees (subjects) and organizations (objects). Berkovich and Eyal (2018) revealed similar findings while exploring the antecedents of help-seeking behavior. Employees who feel psychologically close to their bosses are more inclined to seek aid from them, according to their research. Berkovich and Eyal (2018) go on to say that those who regard their bosses as providers of social support are less likely to suffer from occupational burnout.

According to Perry et al. (2018), remote work may be a source of work-related burnout if it leads to a lack of socio-emotional support. The absence of socio-emotional assistance indicates a growing social distance between the remote worker and the company. As previously stated, social distance is one of the three dimensions of psychological distance as operationalized by Chen and Li (2018). The other two dimensions are time and space distance. Members of remote teams become temporally far from each other when working from various time zones, according to Ocker et al. (2011), and this distance adds to the problems faced by persons working Psychological distance will moderate the association between social anxiety disorder and burnout remotely. According to remote work research, efficient remote work is only possible when adequate communication technology bridges the spatial barrier between people and organizations (van Zoonen et al., 2021). In absence of such ubiquitous technological support, remote working can become a major source of burnout. It is hypothesized based on the evidence presented above.

H4: Psychological distance will mediate the association between social anxiety disorder and burnout.

## 3. Methodology and data collection

According to a McKinsey analysis (McKinsey, 2018), employees working in computing and mathematics are the most likely to be provided remote working opportunities by their employers. Taking this into account, the current study recruited its sample from individuals working for software companies based in Pakistan. Participants for the study were recruited through LinkedIn, the preferred professional social network. The use of social media to attract research participants is becoming more common among social scientists (Darko et al., 2022; Hopkins and Schwanen, 2022).

The current study's sampling methodology is classified as a purposive sampling technique (López, 2022). López (2022) empirically demonstrates that both random and purposive sampling can yield large samples and that both methods are comparable when compared to criteria consistency and unbiased estimators of population parameters. The benefit of adopting purposive sampling in the context of the current study was that it allowed for the selection of persons who had firsthand experience with remote work. The study also used snowball sampling, whereby participants were invited to forward the survey link to other employees working remotely within their organizations or outside of them.

To avoid common method bias, the study adopted a time-lagged design (Podsakoff et al., 2012). Data from the respondents were collected at three different points of time [time 1 (T1), time 2 (T2), and time 3 (T3)]. At T1, data were collected regarding the participants' demographics that included age, year of birth, gender, qualification, experience, and grandfather's name. In addition to the demographics data, the participants were also administered the SAD instrument at T1. At T2, data were collected on moderating variables, namely psychological safety, and workplace attachment orientation. At T3, data were collected on mediating variables, i.e., psychological distance and dependent variable, i.e., burnout. At T1, 670 links were shared, of which 463 responses were received. To control missing values in the data, all items setting were kept as “required”. At T2, following the 4-week lead, the same 463 respondents were again approached to fill in the survey link. The same was repeated for T3 also. Subsequently, self-generated codes (including year of birth and grandfather's name) were used to match the survey across the three time periods. The overall response rate was 69.10%, which is higher than the average response rate reported. Demographic results are presented in Table 1.

**Table 1:** Demographic characteristics.

**Demographic characteristics**	**Frequency**	**Cumulative percentage**
**Age**		
20–30	198	42.7
31–40	242	52.26
41 and above	23	100
**Gender**		
Male	341	73.6
Female	122	100
**Education**		
Certifications	36	7.7
Bachelors	297	64.14
Master or above	130	100
**Experience**		
1–3	242	52.26
4–6	209	45.14
7 and above	12	100

### 3.1. Measures

All factors in this study were operationalized using validated scales from the literature. The questionnaire was handed out in English. To analyze survey items, the survey was pilot tested on eight employees from software companies. According to the findings of the pilot study, respondents had no problem understanding survey items.

*Social anxiety disorder* was measured using 19 items adopted from the study of Mattick and Clarke (1998). The response option for this measure used 5-point Likert scale (0–4), anchors were not at all, slightly, moderately, very, extremely. A sample item includes “I get nervous if I have to speak with someone in authority.” The coefficient alpha value for measure for this study was 0.89. *Psychological Safety* was assessed using 7-item instrument adopted from Edmondson (1999). Sample items are “If you make a mistake in my organization, it is often held against you” and “Members of my organization are able to bring up problems and tough issues.” The coefficient alpha value for this measure is 0.837. *Psychological distance* was measured using a 6-item scale adopted from the study conducted by Chen and Li (2018). A sample item includes “I will protect organizational interests at the cost of my own interests when necessary.” The coefficient alpha value for this measure is 0.68. *Attachment orientation* was measured using a 6-item scale from the study of Rioux (2006). The responses were measured on a 5-point Likert scale where 1 stands for strongly disagree and 5 for strongly agree. The sample item includes “I am attached to my workplace.” The coefficient alpha value for this measure is 0.71. *Burnout* was measured using MBI-GS scale by Schaufeli et al. (1996). The scale consisted of 16 items, and responses were measured on a 7-point Likert scale ranging from 0 for never and 6 for every day. The sample item includes “I feel emotionally drained from my work” and “I feel used up at the end of my work day.” The scale internal consistency value for this measure is 0.875. The detailed results are presented in Tables 2, 3.

**Table 2:** Factor loadings, Cronbach alpha, composite reliability, and AVE.

**Construct**	**items**	**Outer loadings**	**Cronbach alpha**	**CR**	**AVE**
**SAD**			0.89	0.978	0.688
	SAD1	0.932			
	SAD10	0.936			
	SAD 11	0.644			
	SAD12	0.666			
	SAD13	0.866			
	SAD14	0.792			
	SAD15	0.747			
	SAD16	0.850			
	SAD17	0.740			
	SAD18	0.930			
	SAD19	0.948			
	SAD2	0.827			
	SAD3	0.891			
	SAD4	0.891			
	SAD5	0.762			
	SAD6	0.698			
	SAD7	0.864			
	SAD8	0.955			
	SAD9	0.748			
**PD**			0.68	0.808	0.525
	PD3	0.830			
	PD4	0.782			
	PD5	0.449			
	PD6	0.772			
**AO**			0.719	0.569	0.579
	AO1	0.896			
	AO2	0.963			
	AO3	0.936			
	AO4	0.079			
	AO5	0.918			
**PS**			0.837	0.863	0.558
	PS1	0.727			
	PS3	0.792			
	PS4	0.680			
	PS5	0.708			
	PS6	0.818			
**Burnout**			0.875	0.913	0.548
	B1	0.653			
	B2	0.879			
	B3	0.9			
	B4	0.56			
	B5	0.935			
	B6	−0.469			
	B7	0.904			
	B8	0.785			
	B9	0.909			
	B10	0.426			
	B11	0.632			

**Table 3:** Correlation table.

	**SAD**	**ao**	**b**	**pd**	**ps**
SAD					
Ao	0.736				
B	0.747	0.768			
Pd	0.56	0.65	0.525		
Ps	0.199	0.316	0.253	0.13	

## 4. Analyses and results

Data were analyzed using “partial least square approach to structural equation modeling” (PLS-SEM). A variance-based approach was selected for PLS-SEM, and SmartPLS version 3.0 was used. Following Chin's (1998) two-step approach, the measures were first validated and then further tested for hypothesized model. There were a total of five constructs included in the study.

The variables included in the study are as follows: SAD, attachment orientation, psychological distance, psychological safety, and burnout. For the assessment of the measurement model, scale reliability and validity in relation to latent constructs were analyzed (Hair et al., 2017). The convergent validity and internal consistency reliability for model under study with composite reliability and average variance extracted were assessed. The composite reliability of all latent constructs for the model was higher than 0.70 (Hair et al., 2017; Chin et al., 2020). Similarly, Cronbach's alphas were also within the acceptable threshold. The results of measurement model indicate good internal consistency. Furthermore, convergent and discriminant validity was carried out to corroborate the validity of the results (Hair et al., 2011). AVE is executed for all constructs, and all the AVE for all constructs fell within the range of an acceptable threshold of 0.50 (Hair et al., 2011). Further, HTMT criterion was used, and all the values were above 0.85 (Henseler et al., 2015). The structural model was assessed with 5,000 replications using bootstrapping technique, which establishes the discriminant validity of the measurement model (Table 4).

**Table 4:** Test of HTMT.

**Variable names**	**1**	**2**	**3**	**4**	**5**
(1) SAD					
(2) Ao	0.736				
(3) B	0.747	0.768			
(4) PD	0.56	0.65	0.525		
(5) PS	0.199	0.316	0.253	0.13	

Once the psychometric properties of the measurement model are met, then the data are further processed for structural model assessment. In the structural model, proposed hypotheses are tested. The SRMR value is 0.212, which is <0.08 indicating a good model fit. The total effect of the model is presented in Table 5. Table 5 illustrates the direct effect; the results show the presence of a relationship between SAD and burnout (β = 0.716, *t*-value = 24.108), SAD and psychological distance (β = 0.16, *t*-value = 2.749).

**Table 5:** Total effect, direct effect, and indirect effect.

**Paths**	**Path-coefficient (*t*-value)**	**95% confidence intervals**
**Direct effects**		
Attachment orientation -> psychological distance	0.452 (7.756)	0.354 (0.551)
Psychological distance -> burnout	0.445 (12.362)	0.06 (0.181)
Psychological safety -> psychological distance	−0.019 (0.193)	−0.124 (0.196)
Social anxiety order -> burnout	0.716 (24.108)	0.672 (0.772)
Social anxiety order -> psychological distance	0.16 (2.749)	0.064 (0.256)
**Mediation effects**		
Psychological safety × social anxiety order -> psychological distance -> burnout	−0.01 (1.646)	−0.0170 (−0.0220)
Attachment orientation × social anxiety order -> psychological distance -> burnout	−0.004 (0.62)	−0.014 (−0.005)
**Process**		
PS x SAD -> PD -> BO	−0.042 (3.33)	−0.067 (−0.017)
PS -> PD -> BO	0.115 (2.778)	0.03 (0.195)
AO x SAD -> PD -> BO	−0.02 (1.776)	−0.044 (0.002)
AO -> PD -> BO	0.199 (4.093)	0.106 (0.301)
SAD -> PD -> BO	0.224 (4.155)	0.123 (0.337)

## 5. Discussion

As firms investigate the benefits of adopting remote working as an option, it would be useful to understand how remote working might affect various categories of people. The current study looked at how remote working might influence people who suffer from SAD as a subset of employees. The study hypothesized that SAD would increase psychological distance and that this perceived distance would result in burnout. The hypothesis that SAD increases psychological distance (H1) was validated, but the mediation effect of psychological distance in the link between SAD and burnout (H4) was not. The implication is that SAIs are less prone to develop work-related burnout due to the higher psychological distance. There is little prior research to support this conclusion, but anecdotal reports (Packer, 2021) appear to show that those suffering from social anxiety prefer the option of working remotely because it minimizes the frequency of social encounters. This lack of social exposure may actually contribute to the reduction of stress that these employees experienced while working within the physical constraints of their organizations (Brown, 2021) (refer to Table 6).

**Table 6:** Hypothesis table.

**Sr #**	**Hypothesis**	**Accepted/rejected**
H1	SAD will lead to an increased level of psychological distance.	Accepted
H2	Psychological safety will moderate the mediated relationship between SAD and burnout.	Not accepted
H3	Attachment orientation will moderate the mediated relationship between SAD and burnout	Accepted
H4	Psychological distance will mediate the relationship between SAD and burnout.	Not accepted

**Figure 1:**
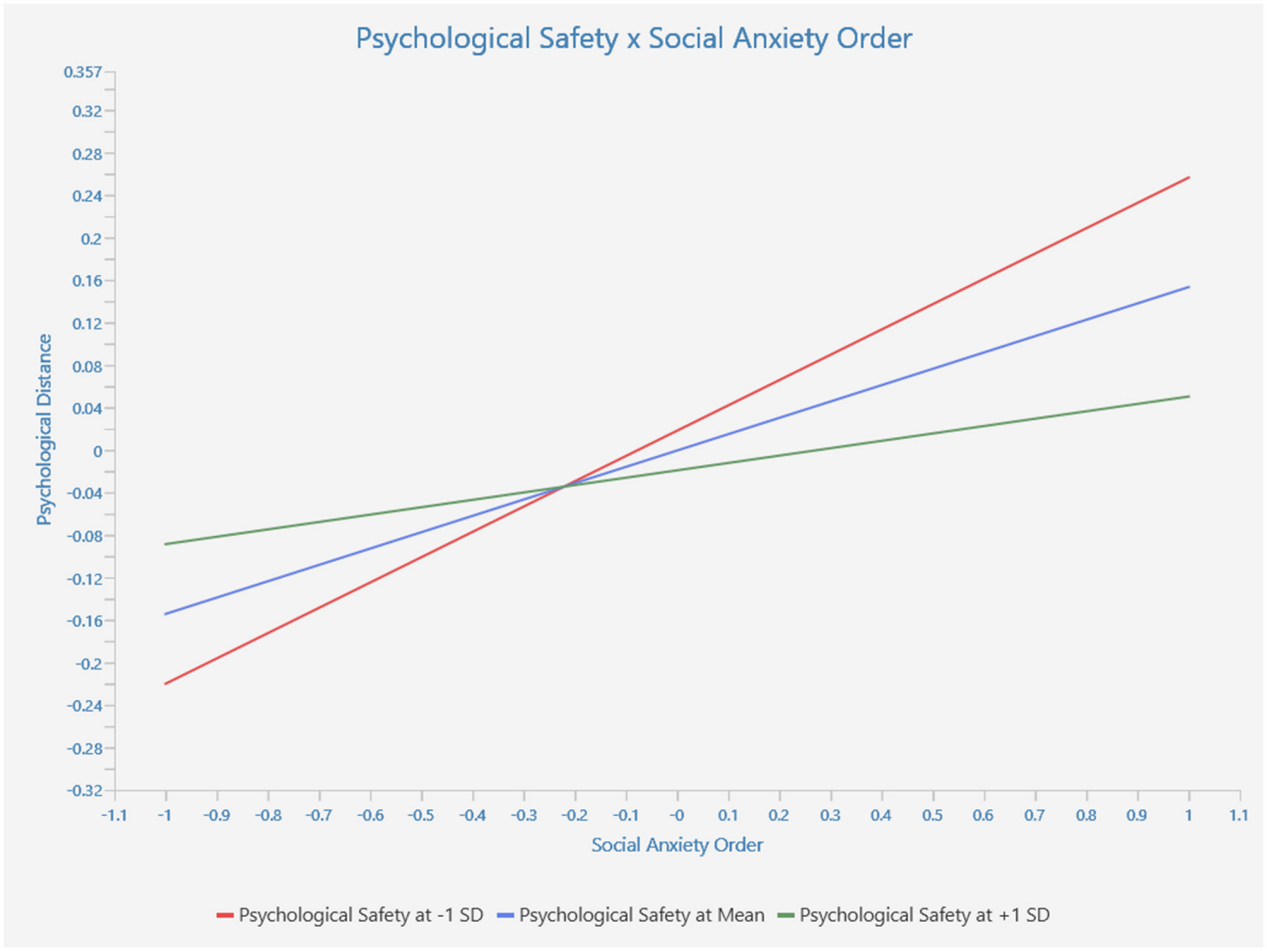
Measurement model.

**Figure 2:**
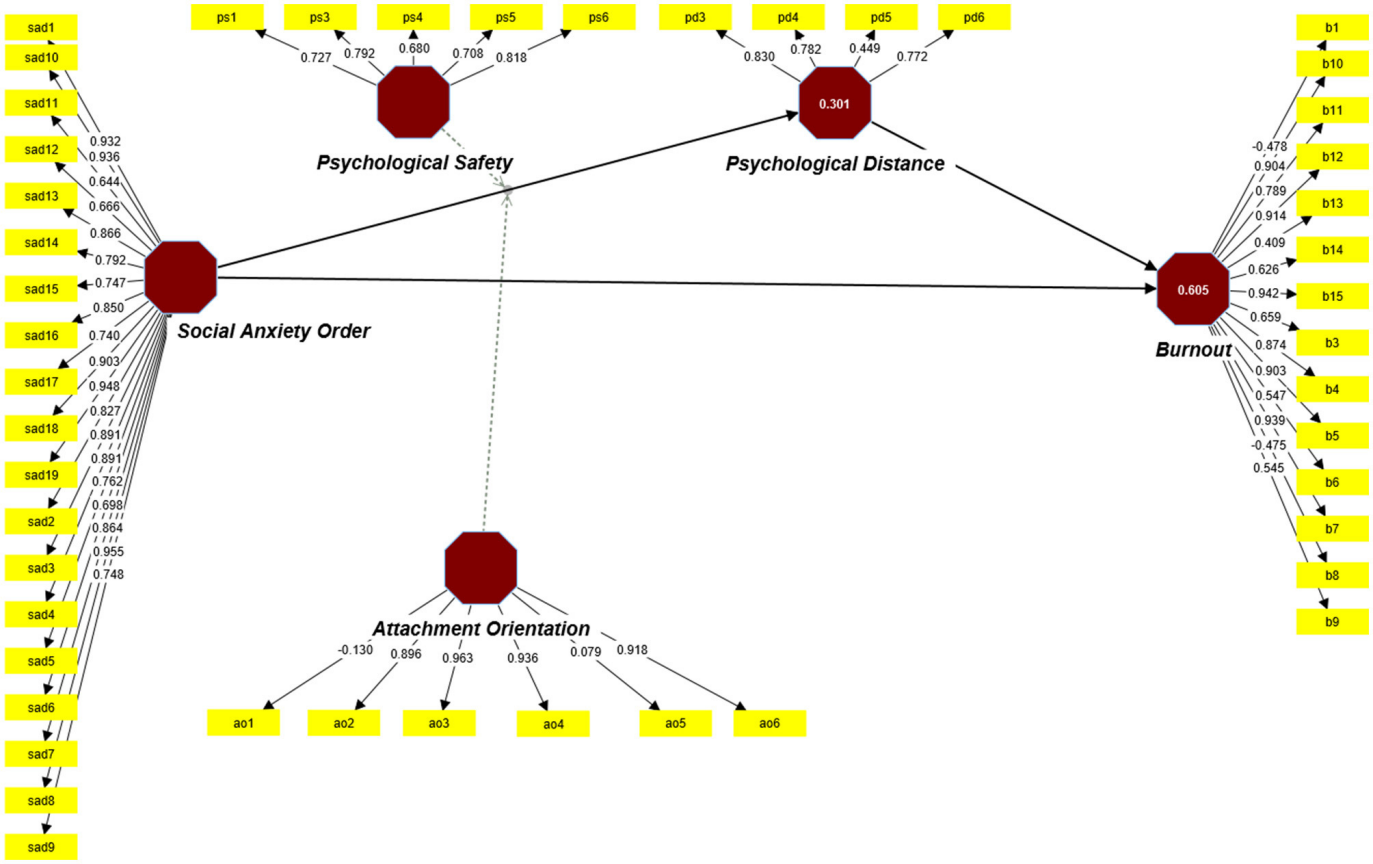
Moderation graph.

The hypothesis that psychological safety would moderate the connection between SAD and psychological distance was rejected in terms of moderation effects (Figure 1). This could be because maintaining a psychologically secure atmosphere online is more challenging than in a physical place (Tkalich et al., 2022), as more effort from the organization is necessary to fulfill this goal (Sjöblom et al., 2022). The hypothesis (H3) revealed support for the moderation effect of workplace attachment orientation. This finding is consistent with a prior study on workplace attachment, which found that a positive attachment orientation toward the organization increases a sense of proximity to the company (see Figure 2).

The study's findings suggest that organizational policymakers who are considering implementing future work-from-home choices or recalling remote workers to physical work should consider viewing their employee pool as a heterogeneous set of distinctive features. Although policies are rarely designed to accommodate individual needs, understanding how the policy may affect diverse groups of people might be beneficial in developing more inclusive outcomes. Future studies could also look into establishing more comprehensive burnout models that are particular to remote working and SAIs. This would be an excellent addition to the literature on remote work and SAD.

### 5.1. Practical implications

This study provides significant insights for researchers and practitioners. Individuals who experience SAD syndrome face difficulties in social interactions, professional settings, have difficulty managing relationship, and experience emotional distress.

Individuals with social anxiety disorder and exhaustion may experience workplace difficulties. Burnout is characterized by depletion, diminished motivation, and cynicism, which can further reduce work output and job satisfaction. Due to fear of social interactions, individuals with a social anxiety disorder may have difficulty forming and maintaining meaningful relationships with others. The emotional exhaustion and cynicism that accompany burnout may impair relationships and diminish the availability of social support networks. Burnout's chronic stress and emotional exhaustion, in conjunction with social anxiety disorder's social concerns and avoidance behaviors, can contribute to a destructive cycle of distress and diminished functioning. Notably, additional research is necessary to understand the complexities of the relationship between social anxiety disorder and exhaustion and their practical implications. Although the cited articles do not explicitly address this connection, they do shed light on the individual characteristics of social anxiety disorder and depletion. To gain a deeper comprehension of this topic, it would be useful to consult additional research on the intersection of social anxiety disorder and depletion. Therapeutic interventions that target attachment-related concerns, such as enhancing attachment security or reducing attachment-related anxiety, may be beneficial. In addition, addressing the perception of psychological distance and working to develop social skills and self-esteem can be crucial components of treatment.

### 5.3. Theoretical implications

Attachment theory is supported by the effect of attachment orientation on the relationship between social anxiety disorder and psychological distance. Attachment theory provides a framework for understanding how experiences affect attachment styles, emotional regulation, and relationship patterns in adulthood. The complex relationship between attachment, social anxiety, and psychological distance is examined by examining these theoretical concepts. The relationship between attachment orientation, social anxiety disorder, and fatigue is consistent with attachment theory, which emphasizes the importance of early interactions in forming attachment patterns and future interpersonal functioning. Attachment theory emphasizes the significance of early relationships in the development of attachment patterns. By conducting additional research into the underlying theoretical processes, one might gain a deeper understanding of the dynamic relationship between attachment, social anxiety, and fatigue.

### 5.4. Limitations and future recommendations

Based on the above discussion, characterizing SAD as a predictor of burnout does not entail enough evidence. As presented in previous literature, antecedents leading to burnout are heterogeneous, and few of them overlap with depression symptoms. The current study is not free from limitations. The data were collected at different points of time but depend on reporting by the respondents. There is a chance of biased estimates. Therefore, future studies should collect data utilizing different methods to avoid issues. The data are collected through purposive sampling. Using other sampling techniques would provide a more effective selection of samples and improve results. Future researches should consider investigating mediating and moderating the role of other organizational values. The moderating effect of leadership styles can also be investigated to measure how servant leadership style or transformational leadership can help in reducing in negative effects of burnout. The moderating role of organizational support and psychological empowerment can also be examined.

## Data availability statement

The raw data supporting the conclusions of this article will be made available by the authors, without undue reservation.

## Ethics statement

Ethical review and approval was not required for the study on human participants in accordance with the local legislation and institutional requirements. Written informed consent from the patients/participants or patients/participants legal guardian/next of kin was not required to participate in this study in accordance with the national legislation and the institutional requirements.

## Author contributions

All authors listed have made a substantial, direct, and intellectual contribution to the work and approved it for publication.
